# Recent Trends and Techniques in Computing Information Intelligence

**DOI:** 10.1155/2016/7168519

**Published:** 2016-06-05

**Authors:** Venkatesh Jaganathan, Balasubramanie Palanisamy, Mariofanna Milanova

**Affiliations:** ^1^University of Atlanta, Atlanta, GA 30097, USA; ^2^Kongu Engineering College, Tamil Nadu 638052, India; ^3^University of Arkansas at Little Rock, Little Rock, AR 72204, USA

Computing information intelligence is considered an extremely important asset to any organization. Techniques are set of management disciplines with the help of computing information that allow organizations to manage their technological fundamentals to create competitive business advantage. Computing information intelligence includes integrated planning, design, optimization, operation, and control of technological products, processes, and services: a better definition would be the management of the use of intelligence for human advantage. Information and computing intelligence sciences employ new technologies to solve client problems through contract research. To address the problems a lot of research efforts are still needed from both academia and industry. This special issue aims to promote research in the area of computing information intelligence in present business scenario.

The article entitled “Information Retrieval and Graph Analysis Approaches for Book Recommendation” proposed a combination of multiple information retrieval approaches for the purpose of book recommendation. Different theoretical retrieval models, probabilistic as InL2 (divergence from randomness model) and language model, are used and we tested their interpolated combination. Graph analysis algorithms such as PageRank have been successful in Web environments. We consider the application of this algorithm in a new retrieval approach to related document network comprised of social links. A series of reranking experiments demonstrate that combining retrieval models yields significant improvements in terms of standard ranked retrieval metrics.

The article entitled “Distilling Big Data: Refining Quality Information in the Era of Yottabytes” targets the utilization of the Fuzzy Bayesian process model to improve the quality of information in Big Data.

The article entitled “Framing a Knowledge Base for a Legal Expert System Dealing with Indeterminate Concepts” describes the development of a negotiation decision support system (the Parenting Plan Support System or PPSS) to support parents in drafting an agreement (the parenting plan) for the exercise of parental custody of minor children after a divorce is granted.

In the article entitled “Priority Based Congestion Control Dynamic Clustering Protocol in Mobile Wireless Sensor Networks,” in order to conserve energy and to avoid congestion during multiclass traffic a novel Priority Based Congestion Control Dynamic Clustering (PCCDC) protocol is developed. Simulation results have proven that packet drop, control overhead, and end-to-end delay are much lower in PCCDC which in turn significantly increases packet delivery ratio, network lifetime, and residual energy when compared with PASCC protocol.

The article entitled “Predicting Defects Using Information Intelligence Process Models in the Software Technology Project” gave the practical applicability of using predictive models and illustrated the use of these models in a project to predict system testing defects, thus helping to reduce residual defects.

The article entitled “Scalable Clustering of High-Dimensional Data Technique Using SPCM with Ant Colony Optimization Intelligence” has been developed to cluster data using high-dimensional similarity based PCM (SPCM), with ant colony optimization intelligence which is effective in clustering nonspatial data without getting knowledge about cluster number from the user. The mountain method is applied for searching approximate centers in the cluster, where the maximum density clusters are located. Though this is efficient clustering, it is checked for optimization using ant colony algorithm with swarm intelligence. Thus, the scalable clustering technique is obtained and the evaluation results are checked with synthetic datasets.

In the article titled “Multicriteria Personnel Selection by the Modified Fuzzy VIKOR Method” personnel evaluation is an important process in human resource management; a fuzzy hybrid multicriteria decision-making (MCDM) model is proposed to personnel evaluation. This model solves personnel evaluation problem in a fuzzy environment where both criteria and weights could be fuzzy sets. The triangular fuzzy numbers are used to evaluate the suitability of personnel and the approximate reasoning of linguistic values. A comparative analysis of results by fuzzy VIKOR and modified fuzzy VIKOR methods is presented. Experiments showed that the proposed modified fuzzy VIKOR method has some advantages over fuzzy VIKOR method. Firstly, from a computational complexity point of view, the presented model is effective. Secondly, compared to fuzzy VIKOR method, it has high acceptable advantage compared to fuzzy VIKOR method.

The article entitled “Traffic and Driving Simulator Based on Architecture of Interactive Motion” proposes an architecture for an interactive motion-based traffic simulation environment. The architecture has been designed to enable the simulation of the entire network; as a result, the actual driver, pedestrian, and bike rider can navigate anywhere in the system. The two traffic flow simulation models interact continuously to update system conditions based on the interactions between actual humans and the fully simulated entities. The implementation of the proposed architecture faces significant challenges ranging from multiplatform and multilanguage integration to multievent communication and coordination.

The article entitled “Proactive Alleviation Procedure to Handle Black Hole Attack and Its Version” proposed an alleviation procedure which consists of timely mandate procedure, hole detection algorithm, and sensitive guard procedure to detect the maliciously behaving nodes. It has been observed that the proposed procedure is cost-effective and ensures QoS guarantee by assuring resource availability, thus making the MANET appropriate for Internet of Things.

Overall, this special issue brought together state-of-the-art research contributions, tutorials, and position papers that address the key aspects of computing information intelligence towards the recent trends and techniques.

*Venkatesh Jaganathan* *Venkatesh Jaganathan* *Balasubramanie Palanisamy* *Balasubramanie Palanisamy* *Mariofanna Milanova* *Mariofanna Milanova*

